# Establishment of Mouse Primed Stem Cells by Combination of Activin and LIF Signaling

**DOI:** 10.3389/fcell.2021.713503

**Published:** 2021-08-05

**Authors:** Mengyi Wei, Yanglin Chen, Chaoyue Zhao, Li Zheng, Baojiang Wu, Chen Chen, Xihe Li, Siqin Bao

**Affiliations:** ^1^State Key Laboratory of Reproductive Regulation and Breeding of Grassland Livestock, Inner Mongolia University, Hohhot, China; ^2^Institute of Animal Genetic Research of Mongolia Plateau, College of Life Sciences, Inner Mongolia University, Hohhot, China; ^3^School of Basic Medical Sciences, Southern Medical University, Guangzhou, China; ^4^Inner Mongolia Saikexing Institute of Breeding and Reproductive Biotechnology in Domestic Animal, Hohhot, China

**Keywords:** primed stem cells, embryonic stem cells, conversion, Activin A, LIF

## Abstract

In mice, embryonic stem cells (ESCs) and epiblast stem cells (EpiSCs) are established from pre- and post-implantation embryos and represent the naive and primed state, respectively. Herein we used mouse leukemia inhibitory factor (LIF), which supports ESCs self-renewal and Activin A (Act A), which is the main factor in maintaining EpiSCs in post-implantation epiblast cultures, to derive a primed stem cell line named ALSCs. Like EpiSCs, ALSCs express key pluripotent genes *Oct4*, *Sox2*, and *Nanog*; one X chromosome was inactivated; and the cells failed to contribute to chimera formation *in vivo*. Notably, compared to EpiSCs, ALSCs efficiently reversed to ESCs (rESCs) on activation of Wnt signaling. Moreover, we also discovered that culturing EpiSCs in AL medium for several passages favored Wnt signaling-driven naive pluripotency. Our results show that ALSCs is a primed state stem cell and represents a simple model to study the control of pluripotency fate and conversion from the primed to the naive state.

## Introduction

Embryonic stem cells (ESCs) are known for their potential of self-renewal and differentiating into different embryonic tissues (Evans and Kaufman, 1981). Pluripotency is temporary and transient *in vivo*, whereas *in vitro* many different states of pluripotent stem cells have been established to model the embryonic stem cells of early embryos (Brons et al., 2007; Tesar et al., 2007; Ying et al., 2008; Joo et al., 2014; Ohinata and Tsukiyama, 2014; Yang J. et al., 2017; Yang Y. et al., 2017). Using leukemia inhibitory factor (LIF) and two inhibitors, Ying et al. (2008) established mouse embryonic stem cells, which are defined as “naive.” These naive mESCs are in a ground state of pluripotency and display a distinct morphology and a more uniform gene expression profile than conventional mESCs maintained in cultures supplemented with serum/LIF and, are capable of producing chimera and germline offspring (Ying et al., 2008). In contrast, human pluripotent stem cells or mouse epiblast stem cells, derived from culture medium containing Activin A (Act A) and bFGF and defined as “primed,” fail to contribute to blastocyst chimera formation although they have the ability to form teratoma (Brons et al., 2007; Tesar et al., 2007).

Extensive efforts have been made to identify approaches able to reverse the two states of pluripotent stem cells and mainly involve either specific culture conditions with different factors or forced “naive” gene expression (Bao et al., 2009; Guo et al., 2009; Guo and Smith, 2010; Okashita et al., 2016; Chen et al., 2018; Du et al., 2018; Pastor et al., 2018; Qiu et al., 2015; Rathjen et al., 1999; Zhou et al., 2010; Tai and Ying, 2013; Yu S. et al., 2020). D’Aniello et al. (2017) proposed the pivotal roles of vitamin C and L-proline in controlling the pluripotency continuum from naive to primed states by affecting global DNA methylation, transcriptional profile, and energy metabolism. Recently, Yu S. et al. (2020) proposed that BMP4 plays an essential role in primed-to-naive transition (PNT) by opening up chromatin loci to activate critical regulators of PNT. These reports demonstrate that the state of pluripotent stem cells can be reversed to some extent by factors in their culture conditions. Two recent studies have suggested that there is a “formative” state in ESCs which is between the “naive” and “primed” state and also presents formative features of human stem cells and horse stem cells (Kinoshita et al., 2020; Yu L. et al., 2020). These two studies describe different culture systems. The first involves the inhibition of the Wnt signaling pathway and the cells named FS cells, while the other culture system is dependent on the Wnt signaling pathway and the cells named XPSCs; however, both cell lines exhibit formative pluripotency features (Kinoshita et al., 2020; Yu L. et al., 2020). All intermediate stem cells, including the formative stem cells (FS cells), XPSCs and rosette-like stem cells (RSCs), encode a higher pluripotent gene expression than EpiSCs and contribute to chimera formation; however, different culture conditions and the unique properties of stem cells still require further exploration (Kinoshita et al., 2020; Neagu et al., 2020; Yu L. et al., 2020).

In this study, we investigated which factors play important roles for establishing pluripotent stem cells from mouse post-implantation embryos. Using a chemically defined medium N2B27 supplemented with Act A and LIF, we successfully derived primed pluripotent stem cells. These pluripotent stem cells were named as ALSCs, like EpiSCs, which expressed pluripotent genes *Oct4*, *Sox2*, *Nanog*, and one inactive X chromosome and contributed to multiple tissues in teratoma but failed to contribute to chimera *in vivo*. The ALSCs were in a primed state, closed to EpiSCs, and able to reverse to naive state with high efficiency by activating Wnt signaling.

## Materials and Methods

### Derivation of ALSCs

Mouse gastrulas were collected from E6.5 pregnant female ICR mice mated with GOF/GFP transgenic male mice with a mixed background. The isolate epiblasts (E6.5) were obtained from gastrulas using a glass needle and were cultured in AL medium. After 5–10 days, outgrowths were minced into several smaller pieces using a glass needle and moved into a fresh AL medium. The colonies, named ALSCs, could stably propagate by Accutase (Life Technology) every 2 days at a ratio of 1:4–1:6, and fresh AL medium was provided every day. The AL medium consisted of Act A (20 ng/ml, R&D systems) and LIF (1,000 U/ml, Millipore) added into a basic N2B27 medium including 50% Neurobasal (Gibco), 50% DMEM/F12 (Gibco), 2 mM GlutaMax (Gibco), 1 × non-essential amino acids (NEAA, Gibco), 1 × penicillin/streptomycin (Gibco), 0.1 mM β-mercaptoethanol (Gibco), and 0.005% (25 mg) bovine serum albumin (BSA; Gibco) supplemented with 0.5 × N2 (Gibco), and 0.5 × B27 (Gibco). All culture dishes were coated with fibronectin [1 mg/ml in phosphate-buffered saline (PBS), Millipore] for at least 0.5 hour (h) before use.

### Conversion of ALSCs to rESCs and EpiSCs to epiALSCs

To convert ALSCs to rESCs, ALSCs were dissociated into single cells using Accutase (Invitrogen) and were plated in CL medium or 2iL medium. The CL medium consisted of N2B27 medium supplemented with 3 μM CHIR99021 and LIF (1,000 U/ml), while the 2iL medium consisted of N2B27 supplemented with 3 μM CHIR99021, 1 μM PD0325901, and LIF (1,000 U/ml). To transform EpiSCs to epiALSCs, EpiSCs were cultured in AF medium supplemented with Act A (20 ng/ml) and bFGF (12 ng/ml, R&D systems), then dissociated with Accutase, and plated in AL medium. LDN193189 (100 nM, Selleckchem) and SB431542 (10 μM, Selleckchem) were also added into the AL medium and the CL medium to inhibit BMP4 and Act A, respectively.

### AP Staining

Before staining, cells were placed in four-well plates, washed with 1 × PBS, and then fixed in 4% paraformaldehyde at room temperature for 30 min. The cells were washed with 1 × PBS again, followed by the addition of AP staining solution. The AP staining solution was prepared as follows: 50 μl sodium nitrite solution was gently mixed with 50 μl FRV-alkaline solution and incubated at 37°C for 3 min; next, 2.25 ml H_2_O and 50 μl naphthol-As-BI alkaline solution were added to the mixture. The staining solution with fixed cells was incubated in the dark overnight.

### Karyotype

The tested cells were incubated with 0.2 μg/ml colchicine supplemented to the culture medium for 2 h, followed by cell dissociation using Accutase; the suspensions were centrifuged at 1,500 rpm for 5 min to collect the tested cells. The cell pellets were gently resuspended in 8 ml 0.075 mol/L KCL (Sigma) and incubated at 37°C in a water bath for 40 min for hypotonic treatment. Fixative liquid (methanol/glacial acetic acid = 3:1) of 1 ml was subsequently added to the resuspended cells and mixed gently, and the solution was then centrifuged at 1,000 rpm for 10 min. After discarding the supernatant, the cells were mixed gently in 8 ml fixative solution and incubated in 37°C water bath for 30 min for cell fixation, which was repeated twice. Then, the resuspended cells in 0.5 ml fixative liquid were dropped onto ice-cold glass slides, which were then dried for 1 h at 70°C in a drying oven. The glass slides were stained in Giemsa (Sigma) for 10 min, washed with distilled water, and then air-dried. The preparations were analyzed by LUCIA Cytogenetics.

### Real-Time Quantitative Polymerase Chain Reaction

Total RNA was extracted by Rneasy Mini Kit (Qiagen), and cDNA was isolated by GoScript Reverse Transcription System (Promega). Real-time quantitative polymerase chain reactions (RT-qPCR) were set up using the SYBR FAST Universal qPCR kit (KAPA). Relative expression values were normalized to *Gapdh* expression, and data was processed using the comparative Ct method. Each experiment was performed with technical triplicates. The primers used are listed in Supplementary Table 1. Significant differences between groups were determined using *t*-test, and *P*-values < 0.05 were considered statistically significant.

### Immunofluorescence

The cells for immunofluorescence assays were washed with PBS, fixed in 4% paraformaldehyde for 30 min at room temperature, and then permeabilized with 0.1% Triton X-100 (Sigma) and 1% BSA in PBS for 30 min. The cells were then incubated with the appropriate primary antibody at 4°C overnight. After the cells were washed three times in 1% BSA and 0.1% Triton X-100 in PBS for 5 min per wash, they were incubated with secondary antibody for 1 h at room temperature in the dark and then washed once for 5 min in 1% BSA and 0.1% Triton X-100 in PBS and twice for 5 min in PBS. The cells were then mounted in Vectashield with DAPI (Vector Laboratories). The samples were observed with a laser microscope (Nikon, Tokyo, Japan). The antibodies used are listed in Supplementary Table 2.

### Flow Cytometry

Cells were dissociated into single cells using Accutase and collected using centrifugation. After washing once with PBS, the cell pellet was resuspended with PBS containing 1–5% KSR, followed by filtration using a cell strainer (FACSAria II, BD Biosciences) to remove large clumps of cells. The cells were then subjected to flow cytometry (FACSAria II, BD Company). The GFP fluorescence intensity was detected in the FITC channel. Data analysis was performed using FlowJo X.10.0.7.

### Generation of Chimera

To generate chimeric embryos, donor cells (12–15 cells/blastocyst) were microinjected into the ICR mice blastocoel cavity using a piezo-assisted micromanipulator attached to an inverted microscope. Following the recovery of the injected blastocysts in KSOM medium (Millipore), the chimeric blastocysts were transplanted into the uterus of pseudopregnant ICR female mice at 2.5 days post-coitus (dpc). The chimeric embryos were collected at E6.5–E13.5.

### Generation of Teratoma

ALSCs were disaggregated using Accutase into small cell clusters and resuspended in PBS with 30% growth factor reduced Matrigel (Corning) and 5 μM Y27632 (Selleckchem). A total of 5 × 10^6^ ALSCs were injected under the epithelium of NOD–SCID mice. The tumors were allowed to develop for 3–5 weeks, then fixed in 4% paraformaldehyde, and processed for paraffin sectioning. Sections were then observed following hematoxylin and eosin staining.

### Transcriptome Analysis

The total RNA of EpiSCs and ALSCs was isolated using Rneasy Mini Kit (Qiagen). cDNA was synthesized from purified RNA templates. The cDNA libraries were sequenced using Illumina HiSeq10 × platform. Paired-end reads were mapped to the mouse reference genome (GRCm38/mm10). The differentially expressed genes were compared between samples with the standard false discovery rate ≤0.005, fold change | log2Ratio| ≥1 by edgeR online.^1^ Principal component analysis, hierarchical clustering analysis, Venn diagram, Pearson’s correlation analysis, and pathway enrichment analysis of differential genes were performed using the OmicShare tools.^2^ Gene Ontology term enrichment analysis was achieved using DAVID.^3^

### Western Blotting

Cells were lysed in lysis buffer (Solarbio) with protease and phosphatase inhibitors for 15 min on ice. The extracts were then centrifuged at 12,000 rpm for 10 min at 4°C. The supernatants were denatured in loading buffer (95°C, 5 min) and collected for further analysis. A total of 25 mg of proteins was separated on 10% Protogel and transferred onto a polyvinylidene difluoride membrane. The membrane was blocked in blocking solution (5% milk powder/TBST solution) for 1 h at 37°C. The membranes were washed three times in 0.01% Tween-20/TBS 1 × (TBST) and followed by incubation at 4°C overnight with primary antibody (diluted in 5% milk powder in TBST solution). The membranes were washed three times in TBST solution and incubated with HRP-conjugated anti-rabbit secondary antibodies (diluted in 2% milk powder in TBST solution) at room temperature for 1 h. Protein was then visualized using X-ray films. The antibodies used are listed in Supplementary Table 2.

### Formation of Embryonic Body

ALSCs and EpiSCs on fibronectin-coated plates were incubated with Accutase for 3 min at 37°C till the colonies were completely disaggregated. The colonies were resuspended in embryoid body (EB) medium: DMEM/F12 + 20% KSR (Gibco) + 1% GlutaMax (Gibco) + 1% NEAA (Gibco) + 1% β-mercaptoethanol (Gibco). The colonies were cultured in suspension for 3 days at a concentration of 1 × 10^5^ cells/ml.

## Results

### Derivation of Primed Stem Cells From Post-implantation Embryos Supported by Act A and LIF

Previously we reported that Act A is an essential factor for maintaining the self-renewal of EpiSCs (Chen et al., 2020; Wu et al., 2020). To test the role of Act A, CHIR99021 (CHIR), and LIF in primed stem cells, we first cultured AFSCs, which are essentially a cell line similar to EpiSCs (Bao et al., 2018) in a chemically defined medium containing Act A with CHIR (AC medium) or LIF (AL medium). We found that the AL medium was able to maintain the pluripotency, while the AC medium failed (data not shown), which is in accordance with the notion that the AC medium supports primitive streak formation (Gadue et al., 2006; Turner et al., 2014; Tsakiridis et al., 2015; Morrison et al., 2016). Subsequently, AFSCs were cultured in AL medium and designated afALSCs (Supplementary Figure 1A). We did not observe any distinctive morphological change when AFSCs were placed into AL medium for more than 10 passages (Supplementary Figure 1B), and afALSCs maintained the characteristics of pluripotency by AP staining and immunofluorescence of NANOG and OCT4 (Supplementary Figures 1B,C). RT-qPCR analysis also revealed a higher expression of *Nanog* and *Klf2* in afALSCs than AFSCs (Supplementary Figure 1D).

**FIGURE 1:**
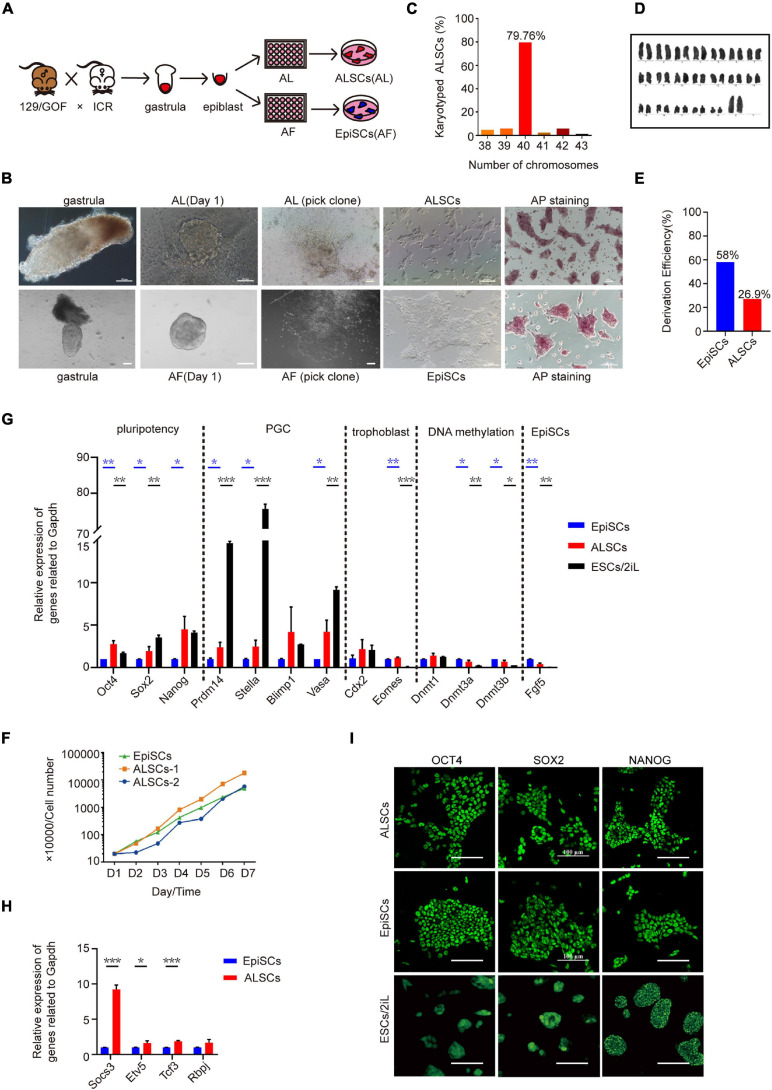
Derivation and characterization of ALSCs. **(A)** Schematic of derivation of ALSCs. **(B)** Bright field image shows the derivation process and AP staining of ALSCs and EpiSCs. Scale bars, 100 μm. **(C)** Distribution of chromosome numbers of ALSCs. **(D)** Karyotype of ALSCs. **(E)** Efficiency of ALSCs and EpiSCs both derived from gastrulas. **(F)** Cell growth curve of EpiSCs and ALSCs. **(G,H)** RT-qPCR of the key genes in ALSCs, EpiSCs, and ESCs/2iL. Error bars indicate three independent biological replicates (mean ± SD). *P < 0.05, **P < 0.001, ***P < 0.0001. **(I)** Immunostaining for pluripotent markers in ALSCs, EpiSCs, and ESCs/2iL. Scale bars, 100 μm.

To further determine the effects of the combination of Act A and LIF, we attempted to derive pluripotent stem cells from mouse gastrulas in AL medium. We first collected E6.5 gastrulas from ICR female mice mated with Oct4ΔPE-GFP reporter (GOF/GFP) male mice and cultured the epiblasts of E6.5 embryos in AL medium (Figures 1A,B). Flat and smooth colonies were selected with a glass needle to mechanically propagate and successfully develop into self-renewal stem cell lines in AL medium over 30 passages (Figure 1B). The stem cells derived in AL medium from gastrulas were named ALSCs and exhibited AP positive (Figure 1B), with normal karyotype (79.76%) (Figures 1C,D), and were GOF/GFP^–^. Furthermore, we evaluated the derivation efficiency of ALSCs to be 27%, which indicated a lower efficiency than 58% of EpiSCs (Figure 1E). These ALSCs displayed the capacity to propagate as quickly as EpiSCs as shown in a growth curve analysis (Figure 1F).

### ALSCs Display Similar Properties With EpiSCs

RT-qPCR analysis in ALSCs revealed high levels of pluripotent gene expression, such as *Oct4*, *Sox2*, and *Nanog*, and low levels of primed pluripotent markers, such as *Fgf5*, compared to EpiSCs (Figure 1G). In addition, we also tested primordial germ cell markers, such as *Prdm14*, *Stella*, and *Vasa*, which were expressed at much lower levels in ALSCs than in ESCs (Figure 1G). Furthermore, in ALSCs markers related to DNA methylation, such as *Dnmt3a* and *Dnmt3b*, showed intermediate levels between those of EpiSCs and ESCs, while the genes related to differentiation, such as *Eomes*, were expressed at a similar level with EpiSCs but were more highly expressed than ESCs (Figure 1G). Interestingly, the formative state markers, *Etv5* and *Tcf3* and *Socs3*, downstream target genes of LIF, showed a higher expression in ALSCs than in EpiSCs, which implied that ALSCs were closer to a formative state than EpiSCs (Figure 1H; Kalkan et al., 2019). Immunofluorescence assays also confirmed the expression of pluripotent markers OCT4, SOX2, and NANOG in ALSCs and the presence of one inactive X chromosome, which was in line with EpiSCs (Figure 1I and Supplementary Figure 1E). Western blotting analysis revealed that P-STAT3 was expressed in ALSCs at an intermediate level between that of ESCs and EpiSCs (Supplementary Figure 2A).

To further understand which factors were important for the maintenance of ALSCs, we cultured ALSCs in medium with Act A or LIF alone (Supplementary Figure 2B). Unexpectedly, ALSCs were unable to sustain self-renewal in the medium supplemented with LIF alone and appeared apoptotic after three passages. In contrast, ALSC-like colonies were sustained in the presence of Act A alone (Supplementary Figure 2B) and continued to self-renew up to passage 10. Moreover, the RT-qPCR results showed that the pluripotent genes *Oct4*, *Sox2*, and *Nanog* were significantly reduced in LIF or Act A-alone medium. Furthermore, *Socs3*, a gene downstream of the LIF pathway, endoderm genes *Gata4* and *Sox17*, and ectoderm-related genes *Pax6* and *Map2* were significantly increased in the LIF medium than in control ALSCs (Supplementary Figures 2C,D). These data suggested that Act A was important but not sufficient to sustain ALSC growth and self-renewal, which is consistent with previous studies (Ashida et al., 2017; Mulas et al., 2017; Chen et al., 2020). We also show that the combination of Act A and LIF could support the pluripotency of ALSCs.

Taken together, our results demonstrated that a chemically defined medium with Act A and LIF was capable of establishing ALSCs from E6.5 gastrulas. We showed that ALSCs were primed pluripotent stem cells that stably express pluripotent markers but display molecular properties similar to those of EpiSCs.

### Developmental and Differentiated Potency of ALSCs *in vivo*

**FIGURE 2:**
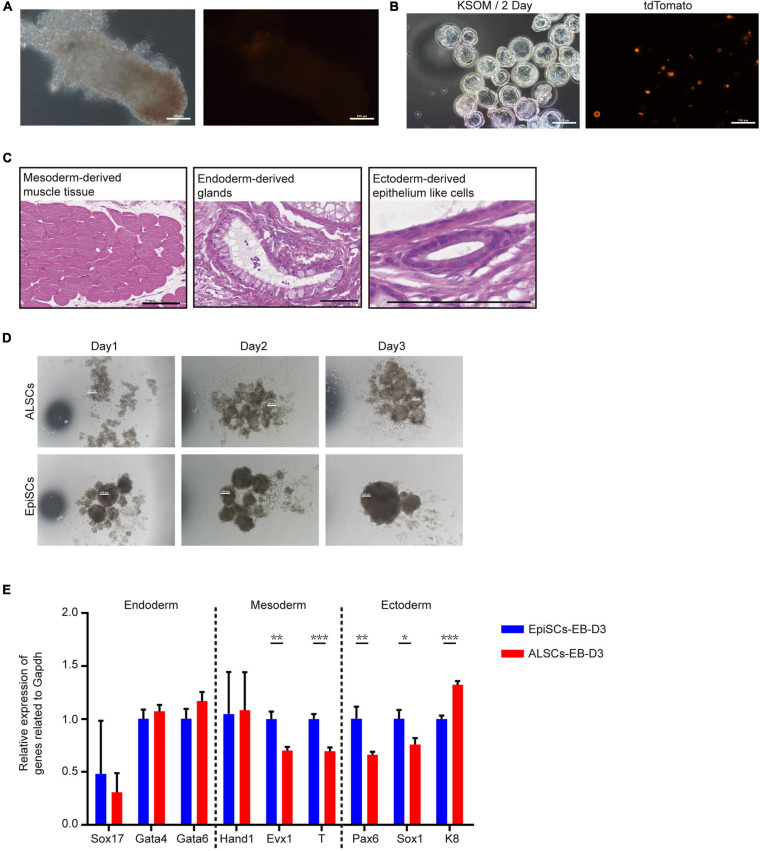
The pluripotency of ALSCs. **(A)** E6.5 chimera collected by injecting ALSCs in host blastocysts. Scale bars, 100 μm. **(B)** E3.5 chimeras generated by injecting ALSCs to eight-cell stage embryos followed culturing in KSOM for 2 days. Scale bars, 100 μm. **(C)** ALSCs contributed to the generation of multiple tissues of germ layer *in vivo* by the teratoma test. Scale bars, 100 μm. **(D)** Morphology of embryoid body induction by ALSCs and EpiSCs for 3 days. Scale bars, 100 μm. **(E)** RT-qPCR of germ layer markers in embryoid bodies of ALSCs and EpiSCs after 3 days of culture. Error bars indicate three independent biological replicates (mean ± SD). *P < 0.05, **P < 0.001, ***P < 0.0001.

To further investigate the pluripotency of ALSCs, we performed chimera tests *in vivo*. ALSCs were transfected with H2B tdTomato plasmid, and 12–15 ALSCs were injected into mouse blastocysts. The chimeric embryos were transplanted in 2.5 dpc pseudopregnant female mice. Unexpectedly, we did not detect any ALSCs with tdTomato fluorescence in the E6.5 chimeras (Figure 2A and Table 1). As ALSCs did not contribute to chimera *in vivo*, we injected eight to 10 ALSCs with tdTomato reporter into eight-cell embryos and then cultured them *in vitro* for 48 h; however, the ALSCs immediately became apoptotic, suggesting that ALSCs could not form chimeras or synchronize with recipient embryo developmental stage (Figure 2B). Teratoma formation was also performed and showed that ALSCs could differentiate to multiple tissue types *in vivo* (Figure 2C and Supplementary Figure 3A). These data suggest that ALSCs were similar to EpiSCs in chimera and teratoma formation (Brons et al., 2007; Tesar et al., 2007). We also compared the embryoid body (EB) formation of ALSCs and EpiSCs for 3 days, and our results showed that the diameter of the EB spheres of ALSCs was smaller than that of EpiSCs (Figure 2D). However, the RT-qPCR analysis showed no distinct difference between the two types of EB (Figure 2E). We further tested the gene expression of the differentiated ALSCs and EpiSCs in N2B27 medium for 6 days by RT-qPCR and obtained similar results (Supplementary Figure 3B). Furthermore, we examined the response of ALSCs to the cytokine cocktail for primordial germ cell induction (Ohinata et al., 2009), but we did not detect GOF/GFP and VASA double-positive cells (Supplementary Figure 3C).

**TABLE 1:** ALSCs do not contribute to E6.5 chimera by injection to blastocysts.

Cell line	Stage of embryo	No. of injected cells	No. of collected embryos	No. of chimeras
ALSCs	E6.5	12–15	12	0

Taken together, these data suggested that the ALSC cell line was similar to EpiSCs for its developmental capacity *in vivo* and *in vitro*.

### Molecular Features of ALSCs

To better understand the molecular features of afALSCs and ALSCs freshly derived from embryos, we first performed RNAseq to analyze the transcriptomes of afALSCs and AFSCs (Supplementary Figure 3D). There were 430 differently expressed genes with 261 up- and 169 downregulated genes in afALSCs. Among the upregulated genes, four distinct gene modules were identified. Module I was related to cytokine-mediated signaling pathways (such as the JAK-STAT cascade), which were stimulated by LIF. Similar to module I, genes from module II were involved in growth hormone signaling pathways. By performing a similar analysis, we also identified two gene modules (modules III and IV) among the genes upregulated in afALSCs. Significant numbers of genes of SMAD protein signal transduction and protein phosphorylation were identified in the conversion of AFSCs to afALSCs. Collectively, these data suggested that the AFSCs are reset in AL medium and present reinforced pluripotency, while the induced afALSCs possess unique molecular features that are distinct from AFSCs (Supplementary Figure 3D).

**FIGURE 3:**
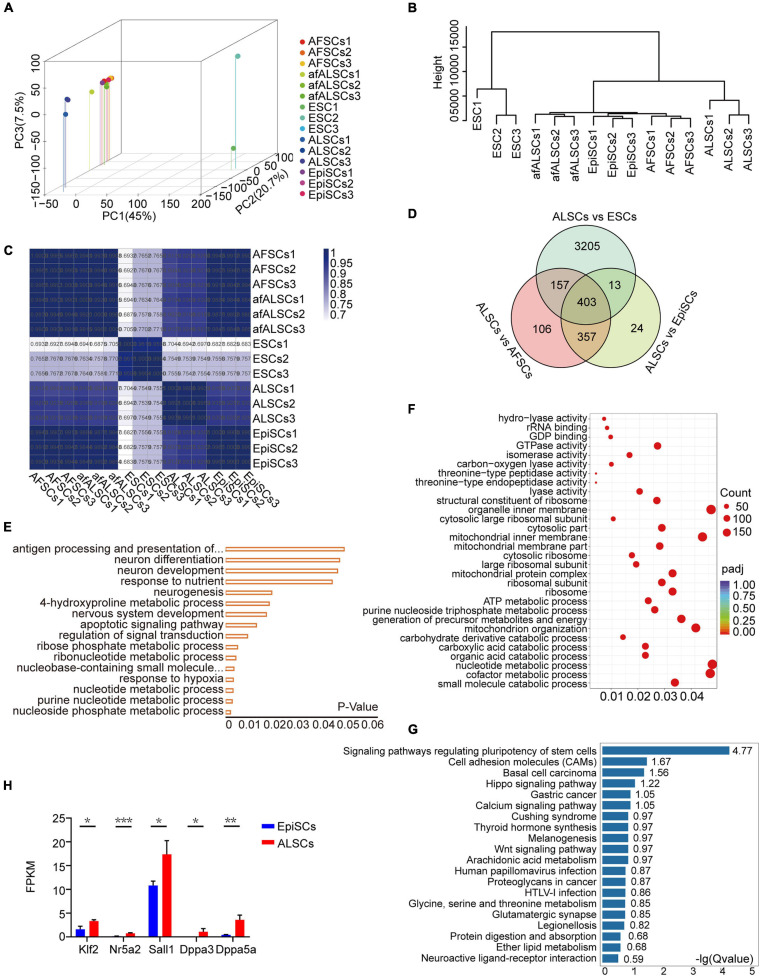
Transcriptomic characteristics of ALSCs. **(A)** Three-dimensional scatter plot based on the principal component analysis of RNA-seq data from three biological replicates. **(B)** Hierarchical clustering of RNA-seq data of ESCs, EpiSCs, ALSCs, afALSCs, and AFSCs. **(C)** The correlation matrix of gene expression was clustered using Pearson correlation. **(D)** Venn diagram showing overlaps of the differential expression gene of ALSCs *vs*. ESCs, ALSCs *vs*. EpiSCs, and ALSCs *vs*. AFSCs. **(E)** Gene Ontology categories significantly enriched in the biological process for 403 genes from the overlap in **(D)**. **(F)** Kyoto Encyclopedia of Genes and Genomes (KEGG) pathway analysis of upregulated genes in 403 genes from the overlap in **(D)**. **(G)** KEGG pathway analysis of upregulated genes in ALSCs *vs*. EpiSCs. **(H)** Pluripotent genes and naive genes were upregulated in ALSCs.

To characterize the molecular features of ALSCs, we assessed the transcriptomes of ALSCs, EpiSCs, and ESCs. Principal component analysis revealed that the global gene expression patterns of ALSCs, afALSCs, AFSCs, and EpiSCs were distinct from ESCs (Figure 3A). Hierarchical cluster analysis demonstrated that afALSCs, AFSCs, and EpiSCs were clustered closely and ALSCs were clustered in the second layer (Figure 3B). This unique clustering of ALSCs was further confirmed by constructing a correlation matrix of gene expression clustered using Pearson correlation coefficients (Figure 3C). We identified 403 genes which were uniquely presented in ALSCs, including *Etv4*, *Lef1*, *Dusp9*, and *Sox3* (Figure 3D). It is interesting to note that the expression of formative markers *Tcf3* in ALSCs suggested that ALSCs were closer to the formative state than EpiSCs, but the low expression of *Otx2* demonstrated that ALSCs did not reside in a formative state (Supplementary Figure 3E; Kinoshita et al., 2020). These characteristic genes were obtained from the intersection of differently expressed genes of ALSCs *vs*. ESCs, ALSCs *vs*. AFSCs, and ALSCs *vs*. EpiSCs. The gene ontology analysis of most upregulated differently expressed genes showed an association with metabolic and neurogenesis processes (Figure 3E). In line with the gene ontology, we used gene set enrichment analysis to reveal the KEGG pathway enrichment for metabolic process (Figure 3F). Consistent with ALSCs being in a state between naive and primed state, most ALSCs *vs*. EpiSCs upregulated differentially expressed genes were related to pathways regulating the pluripotency of stem cells such as *Klf2*, *Nr5a2*, *Sall1*, and *Dppa3* (Figures 3G,H). Indeed *Klf2* and *Nr5a2* are known as naive pluripotency markers, thus our data suggested that ALSCs were closer to the naive state than EpiSCs (Figure 3H).

A recent study suggested that inhibition of Wnt signaling and retinoids by XAV939 and BMS493, respectively, with low concentrations of Act A could capture mouse and human stem cells with formative pluripotent features, defined as FS cells (Kinoshita et al., 2020). Similar to FS cells, ALSCs expressed lower levels of somatic markers, such as *Foxa2* and *T*, and higher levels of pluripotent genes than EpiSCs (Figure 3H and Supplementary Figure 3E). However, ALSCs did not reveal the expected generally low expression of neural lineage markers, such as *Sox11*, *Sox1*, and *Pax6*, which differed from FS cells (Supplementary Figure 3E). Moreover, ALSCs expressed active canonical Wnt signaling pathway genes with a high expression of β*-catenin* and *Tcf3*, and a low expression in *Gsk3*β and *Axin1*, molecules involved in the β*-catenin* destruction complex, which is distinctly different with recent studies (Supplementary Figure 3E; Kim et al., 2013; Kinoshita et al., 2020; Neagu et al., 2020). We also showed that *Otx2* was expressed at a low level in ALSCs, which was also in contrast with previous studies suggesting that *Otx2* played a crucial role in pluripotent stem cells in the intermediate state; however, the specific impact of *Otx2* on ALSCs still needs to be investigated further (Supplementary Figure 3E; Acampora et al., 2013; Kinoshita et al., 2020; Neagu et al., 2020). Interestingly, we found that *E-cadherin* displayed a lower expression in ALSCs than in EpiSCs, which may explain the different morphology of the embryoid body that ALSCs formed (Supplementary Figure 3E).

These results indicated that the ALSCs were primed stem cells with particular molecular features which were similar to EpiSCs.

### Conversion of ALSCs to Naive ESCs

**FIGURE 4:**
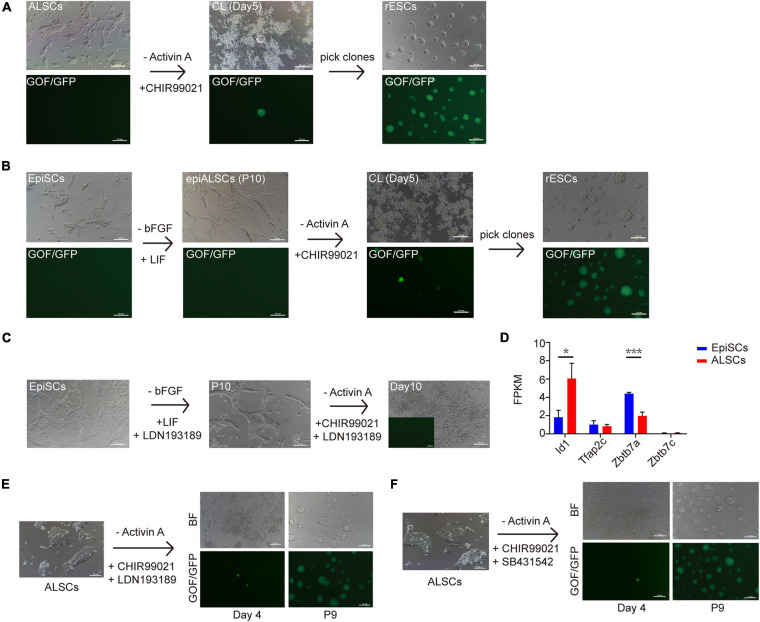
Conversion of ALSCs to rESCs. **(A)** Morphological changes and rESC induction process of ALSCs. Scale bars, 100 μm. **(B)** Morphological changes and rESC induction process of EpiSCs. Scale bars, 100 μm. **(C)** Morphological changes and rESC induction process of EpiSCs supplemented with LDN193189 (BMP4 inhibitor) in AL medium and CL medium. Scale bars, 100 μm. **(D)** The ALSCs maintenance was independent of BMP signaling. Error bars indicate three independent biological replicates (mean ± SD). *P < 0.05, **P < 0.001, ***P < 0.0001. **(E)** Morphological changes and rESC induction process of ALSCs supplemented with LDN193189 (BMP inhibitor) in CL medium. Scale bars, 100 μm. **(F)** Morphological changes and rESC induction process of ALSCs supplemented with SB431542 (Act A inhibitor) in CL medium. Scale bars, 100 μm.

Multiple recent reports have focused on the conversion of primed ESCs to naive ESCs (Chen et al., 2018; Du et al., 2018; Pastor et al., 2018; Yu S. et al., 2020). CHIR is suggested to be a main factor for naive ESCs (Qiu et al., 2015; Choi et al., 2017) as it inhibits the phosphorylation of β-catenin and simulates canonical Wnt signaling. To explore the possibility of conversion from ALSCs to naive ESCs, we cultured ALSCs (GOF/GFP reporter) in medium in which Act A was replaced with CHIR (CL medium) (Figure 4A). After ALSCs were cultured in CL for 4 days, GOF/GFP^+^ colonies were induced, and the individual GOF/GFP^+^ colonies could be maintained as ESCs in morphology over passage 30 (named rESCs) (Figure 4A). Interestingly, ALSCs cultured in 2iL medium (PD0325901, CHIR, and LIF) showed a reduced efficiency of ALSCs converting to rESCs compared to those cultured in CL medium (Table 2). These data suggested that PD0325901 partially blocked the transition procedure, likely because PD0325901 induced DNA damage and hypomethylation in ESCs (Choi et al., 2017). In contrast, EpiSCs began to differentiate or died in cultures containing 2iL or CL medium from AF medium (Act A and bFGF) and failed to convert to rESCs (Table 2). Following the blastocyst injection of rESCs, we could detect reporter expression in E12.5 chimeric embryos and obtained several overt coat color chimeras (Supplementary Figure 4A and Table 3).

**TABLE 2:** Efficiency of rESCs conversed from ALSCs and EpiSCs in CL and 2iL medium.

Cell line	Culture medium	Total cell lines	No. of rESCs	Percentage
ALSCs	CL	6	3	50%
	2iL	6	1	16.7%
EpiSCs	CL	6	0	0%
	2iL	6	0	0%

**TABLE 3:** rESCs contributed to chimeras by injection to blastocysts.

Cell line	Stage of embryo	No. of injected cells	No. of collected embryos	No. of chimeras
rESCs	E12.5	12–15	18	7

As ALSCs but not EpiSCs were converted to rESCs in CL medium, we hypothesized that EpiSCs may be capable of converting to rESC-like cells after culturing in AL medium (called epiALSCs) for several passages (at least 10 passages) (Figure 4B). As expected, epiALSC colonies also developed to become GOF/GFP^+^ when cultured in CL or 2iL for 4 days (Figure 4B), although the transition rate was lower than for ALSCs (Table 4). These results suggested that the successive conversion from EpiSCs to rESCs was accomplished in two stages, with the first step involving the replacement of bFGF in AF medium to LIF and the second step involving the use of CHIR instead of Act A (stage one: AF-AL; stage two: AL-CL).

**TABLE 4:** Efficiency of rESCs conversed from epiALSCs in CL and 2iL medium.

Cell line	Culture medium	Total cell lines	No. of rESCs	Percentage
epiALSCs	CL	5	2	40%
	2iL	5	1	20%

Onishi et al. (2012) proposed that Act A, bFGF, and LIF could induce domed colonies like naive ESCs, but the low cell density of transition cells led to a failed induction of domed colonies. Thus, we next attempted to test ALSC transition from AL to CL medium with low cell densities (Supplementary Figure 4B). The results showed that, when 1,000, 2,000, and 5,000 ALSCs per well were placed into CL medium in six-well plates, ALSCs could form both dome-like and flat-like colonies in CL medium after 12 days. These colonies were all GOF/GFP^–^ until propagated (Supplementary Figure 4C). We next tested whether a single ALSC could convert to rESCs and found that four GOF/GFP^+^ rESCs were obtained (4/96), and similarly these cells did not develop GOF/GFP^+^ clones until propagated (Supplementary Figures 4D,E). These results demonstrated that ALSCs, even at a low density or as single cells, were capable of transitioning to GOF/GFP^+^ clones in CL medium and that the transition cell density did not impact on the induction of naive-like colonies, which is different with the system proposed by Onishi et al. (2012).

Yu S. et al. (2020) recently proposed that BMP4 was essential for primed-to-naive transition (PNT). Thus, we investigated whether BMP4 was essential for our transition system. We first chose two EpiSC lines which successfully reversed to rESCs by two steps and then cultured them in AL medium (stage one) supplemented with LDN193189, an inhibitor of BMP4. EpiSCs successfully reverted to epiALSCs but could not induce GOF/GFP^+^ colonies in CL medium (stage two) with the addition of BMP4 inhibitors (Figure 4C). This suggested that endogenous BMP4 was essential for the process of reversing EpiSCs to rESCs, which was consistent with the studies of Yu S. et al. (2020). However, we focused on the expression of BMP downstream genes identified by RNAseq and found that, except for *Id1*, BMP targets such as *Tfap2c* and *Zbtb7* families were rarely expressed or steadily expressed in ALSCs (Figure 4D). Thus, we directly cultured ALSCs, from AL medium to CL medium supplemented with LDN193189, and found that GOF/GFP^+^ colonies were induced after 4 days and pure GOF/GFP^+^ colonies could be sustained to passage19 (Figure 4E). Moreover, we also added SB431542 to CL medium to culture ALSCs in order to determine whether Act A was essential for the conversion from ALSCs to rESCs. Interestingly, the GOF/GFP^+^ colonies were also produced in 4 days (Figure 4F).

Thus, our data showed that endogenous BMP4 was essential for conversion from EpiSCs to rESCs, as the inhibition of BMP4 resulted in the failure to convert to rESCs. However, our transition system from ALSCs to rESCs does not require endogenous BMP4 or Act A, which are mechanisms that require further study.

### Epigenetic Changes in ALSCs

**FIGURE 5:**
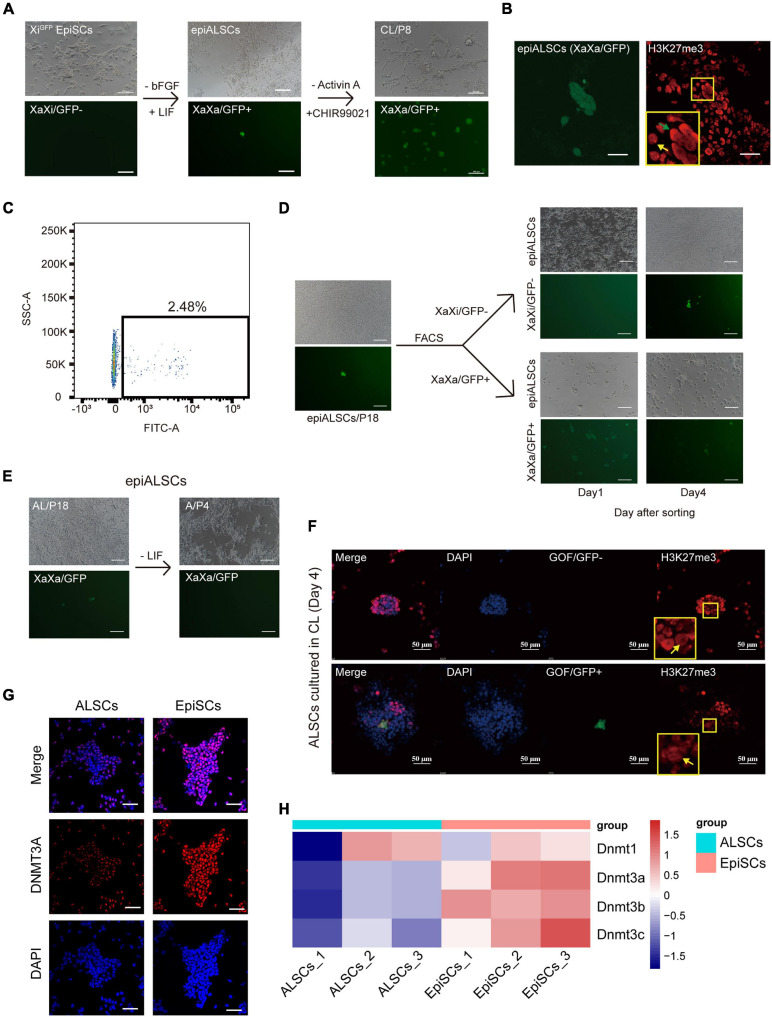
Conversion of Xi^GFP^ EpiSCs to rESCs. **(A)** Morphological changes and rESC induction process of Xi^GFP^ EpiSCs. Scale bars, 100 μm. **(B)** Immunostaining of H3K27me3 in epiALSCs. Scale bars, 50 μm. **(C)** Fluorescence-activated cell sorting analysis of epiALSCs, GFP^+^ cells were Xa^GFP^ epiALSCs. **(D)** Morphological changes and X chromosome change process from isolated XaXa-epiALSCs and XaXi-epiALSCs. Yellow arrow shows XaXi epiALSCs, green arrow shows XaXa epiALSCs. Scale bars, 100 μm. **(E)** Morphological changes and X chromosome inactivation process of epiALSCs placed in Act A-alone medium for four passages. Scale bars, 100 μm. **(F)** Immunostaining of H3K27me3 with GOF/GFP in ALSCs after having been placed in CL medium for 4 days. Yellow arrows show XaXa epiALSCs. Scale bars, 50 μm. **(G)** Immunostaining of DNMT3A in ALSCs and EpiSCs. Scale bars, 50 μm. **(H)** Heat map showing the scaled expression of DNA methylation-related genes.

X chromosome inactivation (Xi) is a major epigenetic change occurring between rESCs and EpiSCs. We first analyzed the X chromosome activation state of ALSCs using Xi^GFP^ EpiSCs, in which the GFP transgene is located exclusively on the Xi, and the reactivation of Xi could be monitored by its GFP expression (Gillich et al., 2012). We observed a stable Xi and did not detect any GFP^+^ cells in Xi^GFP^ EpiSCs (Figure 5A). However, when we cultured these Xi^GFP^ EpiSCs in AL medium, we observed a small number of GFP^+^ cells, which was also confirmed by immunostaining of H3K27me3 (Figure 5B). Flow cytometry analysis showed 2.48% epiALSCs exhibited XaXa (Figure 5C). We next isolated GFP^+^ epiALSCs and GFP^–^ epiALSCs by fluorescence-activated cell sorting to purify the XaXa epiALSCs subset. Unexpectedly, both isolated GFP^+^ and GFP^–^ epiALSCs propagated stably and maintained XaXa and XaXi, respectively, in the first 2 days but quickly reverted to the mixed state at day 4 (Figure 5D), revealing that the AL medium promoted silenced X chromosome reactivation in a few of the epiALSCs, while most sustained a stable Xi^GFP^. Since Act A was capable of sustaining the ALSC pluripotency, we next focused on whether Act A could maintain the reactivation of silenced X chromosome in epiALSCs. Subsequently, we cultured epiALSCs in medium containing Act A alone and observed that, within the first four passages, epiALSCs lost the ability to re-activate the silenced X chromosome (Figure 5E). It is worthy to note that a minority of cells with stable X reactivation was observed in AL medium, which suggested that the combination of Act A and LIF could have a positive effect on X chromosome reactivation.

Next, we examined the impact of CHIR and LIF on X chromosome reactivation during the reversion of ALSCs to rESCs in CL medium. We found that GOF/GFP^+^ colonies started to emerge when ALSCs were cultured in CL medium for 4 days. All GOF/GFP^+^ colonies exhibited XaXa, but some XaXa colonies did not activate GOF/GFP, suggesting that silenced X chromosome reactivation occurred before GOF/GFP activation (Figure 5F), which is in line with the findings of Yu S. et al. (2020). Because DNA methylation is involved in X chromosome inactivation (Sado et al., 2004; Zvetkova et al., 2005; Schmidt et al., 2012; Auclair et al., 2014), we further explored whether DNA methylation levels were altered in ALSCs compared to EpiSCs. We examined the protein expression of the DNA methyltransferase, *Dnmt3a*. The protein expression of DNMT3A by immunofluorescence differed greatly in ALSCs and EpiSCs, which exhibited a sporadic expression in ALSCs but was expressed almost throughout the nucleus in EpiSCs (Figure 5G). As expected, RNA-seq analysis revealed that DNA methyltransferases *Dnmt1*, *Dnmt3a*, *Dnmt3b*, and *Dnmt3c* were significantly downregulated in ALSCs (Figure 5H).

Our data suggested that CHIR was critical for promoting X chromosome reactivation, and in combination with LIF, it drives the conversion to rESCs from primed state stem cells. Our data also indicate that ALSCs possess the plasticity of Xi reactivation, which was supported by the low expression of DNA methyltransferases.

## Discussion

Our findings showed that the AL medium could establish primed pluripotent stem cells from mouse gastrulas and that ALSCs were similar to EpiSCs with unique molecular features. We further demonstrated that ALSCs were able to convert to naive ESCs by activating Wnt signaling, and the AL medium also prompts EpiSCs to transition to an intermediate stage of the naive state from the primed state. Furthermore, our findings showed Act A and LIF were both important for maintaining the unique primed state, as the expression of pluripotent genes decreased in the medium supplemented with either Act A or LIF alone.

In fact, post-implantation epiblasts (E5.0–E6.0) reside in more states, and there is no clear common view on what transcriptional and epigenetic features they may have. Recently, Neagu et al. (2020) showed that LIF could support intermediate pluripotent stem cells for self-renewal. In here we thought that ALSC is in an intermediate state between naive and primed states, LIF push epiblast cells to reversed naive state (Bao et al., 2009), and Act A supports epiblast development (Brons et al., 2007; Tesar et al., 2007). The combination of LIF and Act A was applied to the epiblast to derive unique stem cells, unlike EpiSCs which are activated by Act A and bFGF, displaying a gene signature closer to that of the anterior epiblast of a late-gastrula stage embryo (E7.5) (Kojima et al., 2014). Compared to the culture medium of FS cells and RSCs (Kinoshita et al., 2020; Neagu et al., 2020), the AL medium does not contain inhibitors of the canonical Wnt signaling pathway. Our study showed that canonical Wnt signaling was activated in ALSCs by endogenous stimuli, suggesting that ALSCs maintain a self-renewal capacity independently of the inhibition of Wnt signals, which was different from FS cells and RSCs (Supplementary Figure 3E). However, Wnt signaling plays a critical role in XPSCs having formative features (Yu L. et al., 2020). The effect of β-catenin on ALSCs needs to be further explored, as a recent study demonstrated that turning Wnt signaling on or off could establish the same formative features found in stem cells. FS cells and RSCs utilize a retinoid inhibitor and a MAPK inhibitor, respectively, to suppress differentiation (Kinoshita et al., 2020; Neagu et al., 2020); however, this differs from the AL medium which requires Act A, a component of Nodal signaling, and promotes the differentiation of ESCs.

Notably, neural lineage markers are generally highly expressed in ALSCs at the RNA level, but ALSCs still maintain EpiSC-like colonies and obtain the ability of converting to rESCs, which may illustrate that the inhibition of neural ectoderm differentiation is unnecessary in ALSCs. We hypothesized that endogenous Wnt and MAPK signaling maintained a balanced state in ALSCs, different with FS cells and RSCs, which inhibit Wnt signaling. Importantly, ALSCs efficiently reversed to rESCs when activated by Wnt signaling whereby FS cells failed to reverse to ESCs (Kinoshita et al., 2020). Moreover, instead of producing PGCLCs by FS cells and XPSCs (Kinoshita et al., 2020; Yu L. et al., 2020), ALSCs only induced GOF/GFP^+^ cells; this led us to speculate that ALSCs converted to rESCs rather than generated PGCLCs. From the discussion above, we propose that ALSCs are more similar to a primed state rather than a formative state; nonetheless, the mechanism whereby the AL medium and Act A and LIF cooperate with each other to sustain ALSC self-renewal still requires more investigation.

Compared to the transition system reported by Yu S. et al. (2020), our system did not rely on BMP4, as the known targets of BMP4 were merely expressed independently of *Id1*, which was increased in ALSCs. Moreover, the inhibition of BMP4 did not impact on the capacity of ALSCs to convert to rESCs but influenced the conversion of EpiSCs to epiALSCs, suggesting that endogenous BMP4 did play a vital role during conversion to epiALSCs from EpiSCs. As the transition from the AF medium to the AL medium only required a change in bFGF supplementation to LIF, we considered that the inhibition of BMP4 may be involved in LIF signaling and conversion of EpiSCs to epiALSCs. In addition, the effects of LIF on ALSCs were confirmed by the higher expression of P-STAT3 detected in ALSCs than EpiSCs, illustrating that the Jak/Stat pathway was activated in ALSCs. The impact of LIF signaling in ALSCs could represent the focus of a future investigation. These results imply that endogenous BMP4 was not essential for our transition system and that distinct mechanisms may be involved in these two transition systems (Yu S. et al., 2020).

We showed that ALSCs could enter a primed-like state, which is different from the reported XPSCs, FS cells, and RSCs (Kinoshita et al., 2020; Neagu et al., 2020; Yu L. et al., 2020). We assumed that this intermediate state may represent a state of equilibrium between the two states as what occurs *in vivo*, with the ephemeral state between E4.5 and E6.5. In addition, epiALSCs also exhibited several cells (2.48%) presenting the reactivation of the X chromosome. Therefore, the AL medium for the middle state may include properties from both the primed state and the naive state. Furthermore, ALSCs in the intermediate state may be heterogeneous and maintain a balance between the naive and primed states. Based on our X chromosome reactivation data, we suggest that X chromosome reactivation may not be the main feature of this intermediate state, but rather the chromatin accessibility in this stage may prompt the cells to convert to a naive state more easily, a mechanism which still requires further investigation.

Like EpiSCs, ALSCs failed to contribute to chimera formation despite expressing pluripotent genes and alkaline phosphatase. We speculate that ALSCs are more similar to EpiSCs and exhibit a more compatible embryo stage similar to the post-implantation stage but unlike the cleavage stage of the embryo or blastocyst. Since we identified a higher expression of pluripotent genes and formative genes than in EpiSCs, ALSCs may represent a primed state that can easily revert to ESCs. However, germ layer genes like *Eomes* were also highly expressed in ALSCs, indicating that the detailed mechanisms involved warrant further investigation.

In summary, the AL medium allows to establish ALSCs from post-implantation embryos. ALSCs possess unique molecular properties, propagate robustly in long-term culture, and share a status which could convert to naive state ESCs, thus offering new opportunities to study the control of cell fate.

## Data Availability Statement

All the sequencing data were deposited in the NCBI, Gene Expression Omnibus (GEO) under accession number GSE156673.

## Ethics Statement

The animal study was reviewed and approved by Institutional Animal Care and Use Committee at Inner Mongolia University.

## Author Contributions

SB conceived the idea for this project and designed and conducted the experiments. MW and YC wrote the manuscript with help from all the other authors. MW, YC, CZ, and LZ performed the experiments and analyzed data under the supervision of SB. All the authors read, accepted, and approved the final manuscript.

## Conflict of Interest

The authors declare that the research was conducted in the absence of any commercial or financial relationships that could be construed as a potential conflict of interest.

## Publisher’s Note

All claims expressed in this article are solely those of the authors and do not necessarily represent those of their affiliated organizations, or those of the publisher, the editors and the reviewers. Any product that may be evaluated in this article, or claim that may be made by its manufacturer, is not guaranteed or endorsed by the publisher.

## Footnotes

1
http://www.omicshare.com/tools/Home/Soft/diffanalysis

2
www.omicshare.com/tools

3
http://david.abcc.ncifcrf.gov/
