# Supplementary material for: Establishment of Mouse Primed Stem Cells by Combination of Activin and LIF Signaling
Source: Front Cell Dev Biol. 2021 Aug 5;9:713503. doi: 10.3389/fcell.2021.713503 (PMC8375391; doi:10.3389/fcell.2021.713503)
Supplement: Supplementary file 1 [file Data_Sheet_1.docx]

**Supplementary Material**

**Supplementary Table 1 Primer sequences**

| **qPCR primers** | | | |
| --- | --- | --- | --- |
| **Gene name** | **Forward primer** | **Reverse primer** |  |
| Oct4 | GCTTGGGCTAGAGAAGGATGTG | TGGCGCCGGTTACAGAAC |  |
| Sox2 | CATGAGAGCAAGTACTGGCAAG | CCAACGATATCAACCTGCATGG |  |
| Nanog | AAACCAGTGGTTGAAGACTAGCAA | GGTGCTGAGCCCTTCTGAATC |  |
| Prdm14 | CCTGAACAAGCACATGAGA | TGCACTTGAAGGGCTTCTCT |  |
| Stella | AGGCTCGAAGGAAATGAGTTTG | TCCTAATTCTTCCCGATTTTCG |  |
| Blimp1 | GAGGATCTGACCCGAATCAA | CATGGAGGTCACATCGACAC |  |
| Vasa | TATGTGCCTCCCAGCTTCAGTA | CTGGATTGGGAGCTTGTGAAGA |  |
| Cdx2 | CCTGCGACAAGGGCTTGTTTAG | TCCCGACTTCCCTTCACCATAC |  |
| Fgf5 | AAACTCCATGCAAGTGCCAAAT | TCTCGGCCTGTCTTTTCAGTTC |  |
| Dnmt1 | TGAGGAAGGCTACCTGGCTA | ACAACCGTTGGCTTTTTGAG |  |
| Dnmt3a | GACTCGCGTGCAATAACCTTAG | GGTCACTTTCCCTCACTCTGG |  |
| Dnmt3b | CTCGCAAGGTGTGGGCTTTTGTAAC | CTGGGCATCTGTCATCTTTGCACC |  |
| Eomes | CGGCAAAGCGGACAATAACA | GGAGCCAGTGTTAGGAGATTC |  |
| Socs3 | ATTCACCCAGGTGGCATCAG | GCCAATGTCTTCCCAGTGTT |  |
| Etv5 | GCTGTGGCAGTTCCTTGTC | TTCTTCTGGATACCCCAACG |  |
| Tcf7l1 | TGGTTTCCGGCATCCTTAC | CCACCATGTGTGGGAACC |  |
| Rbpj | AGTCTTACGGAAATGAAAAACGA | CCAACCACTGCCCATAAGAT |  |
| Sox17 | GTCAACGCCTTCCAAGACTTG | GTAAAGGTGAAAGGCGAGGTG |  |
| Gata4 | TTCCTCTCCCAGGAACATCAAA | GCTGCACAACTGGGCTCTACTT |  |
| Gata6 | TGCTGGAAATTGCAACAAACC | GTCACGTGGTACAGGCGTCA |  |
| Hand1 | TCAAAAAGACGGATGGTGGT | GCGCCCTTTAATCCTCTTCT |  |
| Evx1 | CCAGTGACCAGATGCGCCGATAC | TCCTTCATGCGCCGGTTCT |  |
| T | GGACCTCGGATTCACATCGT | TTCTTTGGCATCAAGGAAGG |  |
| Pax6 | GCAGATGCAAAAGTCCAGGTG | CAGGTTGCGAAGAACTCTGTTT |  |
| Sox1 | GGCCGAGTGGAAGGTCATGT | TCCGGGTGTTCCTTCATGTG |  |
| K8 | TCCATCAGGGTGACTCAGAAA | CCAGCTTCAAGGGGCTCAA |  |
| Lefty1 | TGGACAAGGCTGATGAGGAA | TGGCATGGCTGTGTTGTAGC |  |
| Klf2 | GAGCCTATCTTGCCGTCCTTT | CACGTTGTTTAGGTCCTCATCC |  |
| K18 | CAGCCAGCGTCTATGCAGG | CTTTCTCGGTCTGGATTCCAC |  |
| Map2 | AAAGTTGCCTCCAGTTCCATTT | TCTTTGATTCCGTGGGCATTT |  |
| Gapdh | ACCACAGTCCATGCCATCAC | TCCACCACCCTGTTGCTGTA |  |

**Supplementary Table 2 Antibody list**

| **Reagent** | **Source** | **Identifier** | **Dilution Ratio** |
| --- | --- | --- | --- |
| **Antibodies for IF** |  |  |  |
| Mouse monoclonal OCT4 | BD Biosciences | 611203 | 1:200 |
| Goat polyclonal SOX2 | Santa Cruz | Sc-17320 | 1:200 |
| Rat monoclonal NANOG | eBioscience | 14-5761 | 1:500 |
| Rabbit polyclonal H3K27me3 | Millipore | 07-449 | 1:500 |
| Alexa Fluor 488 donkey anti-mouse lgG | Invitrogen | A21202 | 1:500 |
| Alexa Fluor 488 donkey anti-goat lgG | Invitrogen | A11055 | 1:500 |
| Alexa Fluor 488 donkey anti-rat lgG | Invitrogen | A21208 | 1:500 |
| Alexa Fluor 568 donkey anti-rabbit lgG | Invitrogen | A10042 | 1:500 |
| **Antibodies for WB** |  |  |  |
| Rabbit anti-β-ACTIN | Cell signaling | 5125 | 1:1000 |
| Rabbit anti-Phospho-STAT3 | Cell signaling | D3A7 | 1:1000 |
| Rabbit anti-STAT3 | Cell signaling | 79D7 | 1:1000 |
| HRP-conjugated Goat anti-Rabbit IgG | Absin | Abs20002A | 1:5000 |

**Supplementary Figure 1 Characterization of afALSCs**

1. Schematic of derivation of afALSCs.
2. Morphology and AP staining of AFSCs and afALSCs. Scale bars, 100 μm.
3. Immunofluorescence staining for OCT4 and NANOG in AFSCs and afALSCs. Scale bars, 50 μm.
4. Quantitative RT-qPCR of *Nanog* and *Klf2* in AFSCs and afALSCs.
5. Immunofluorescence of OCT4 and H3K27me3 in ALSCs and EpiSCs. Scale bars, 100 μm.

**Supplementary Figure 2 Characterization of ALSCs cultured in Act A and LIF alone**

1. Western blotting of analysis of STAT3 and P-STAT3 in EpiSCs, ALSCs and ESCs/2iL.
2. Morphology of ALSCs cultured in Act A and LIF alone respectively, and AP staining of ALSCs in Act A. Scale bars, 100 μm.
3. Quantitative RT-qPCR of pluripotent genes, LIF downstream target gene *Socs3* and germ layer markers in ALSCs and ALSCs cultured in medium containing Act A alone. Error bars indicate three independent biological replicates (mean±SD). **P*< 0.05, ***P*< 0.001, ****P*< 0.0001.
4. Quantitative RT-qPCR of pluripotent genes, LIF downstream target gene *socs3* and germ layer markers in ALSCs and ALSCs cultured in LIF alone medium. Error bars indicate three independent biological replicates (mean ±SD). **P*< 0.05, ***P*< 0.001, ****P*< 0.0001.

**Supplementary Figure 3 Features of afALSCs and ALSCs**

1. The NOD-SCID mouse with subcutaneous injection of ALSCs.
2. Quantitative RT-qPCR of germ layer genes of EpiSCs and ALSCs after culturing in N2B27 for 6 days.
3. ALSCs and EpiLCs were performed germcell induction. Scale bars, 50 μm.
4. Heatmap of AFSCs and afALSCs.
5. Quantitative RT-qPCR of germ layer genes and Wnt signaling related genes in EpiSCs and ALSCs. Error bars indicate three independent biological replicates (mean ±SD). **P*< 0.05, ***P*< 0.001, ****P*< 0.0001.

**Supplementary Figure 4 Role of low ALSCs concentration on conversion of rESCs**

1. rESCs contributed to chimaeras (E12.5) and germline by injecting rESCs to blastocysts and a chimeric pup generated by injecting rESCs in ICR blastocysts. Scale bars, 100 μm.
2. Schematic of low ALSCs concentration in conversion to rESCs.
3. Morphological changes and GOF/GFPrESCs conversed under low ALSCs concentration. Scale bars, 100 μm.
4. Schematic and morphology of a single ALSC converted to rESCs. Scale bars, 100 μm.
5. Conversion rate of single ALSC to rESCs.
