# Supplementary figures and images for: Establishment of Mouse Primed Stem Cells by Combination of Activin and LIF Signaling
Source: Front Cell Dev Biol. 2021 Aug 5;9:713503. doi: 10.3389/fcell.2021.713503 (PMC8375391; doi:10.3389/fcell.2021.713503)

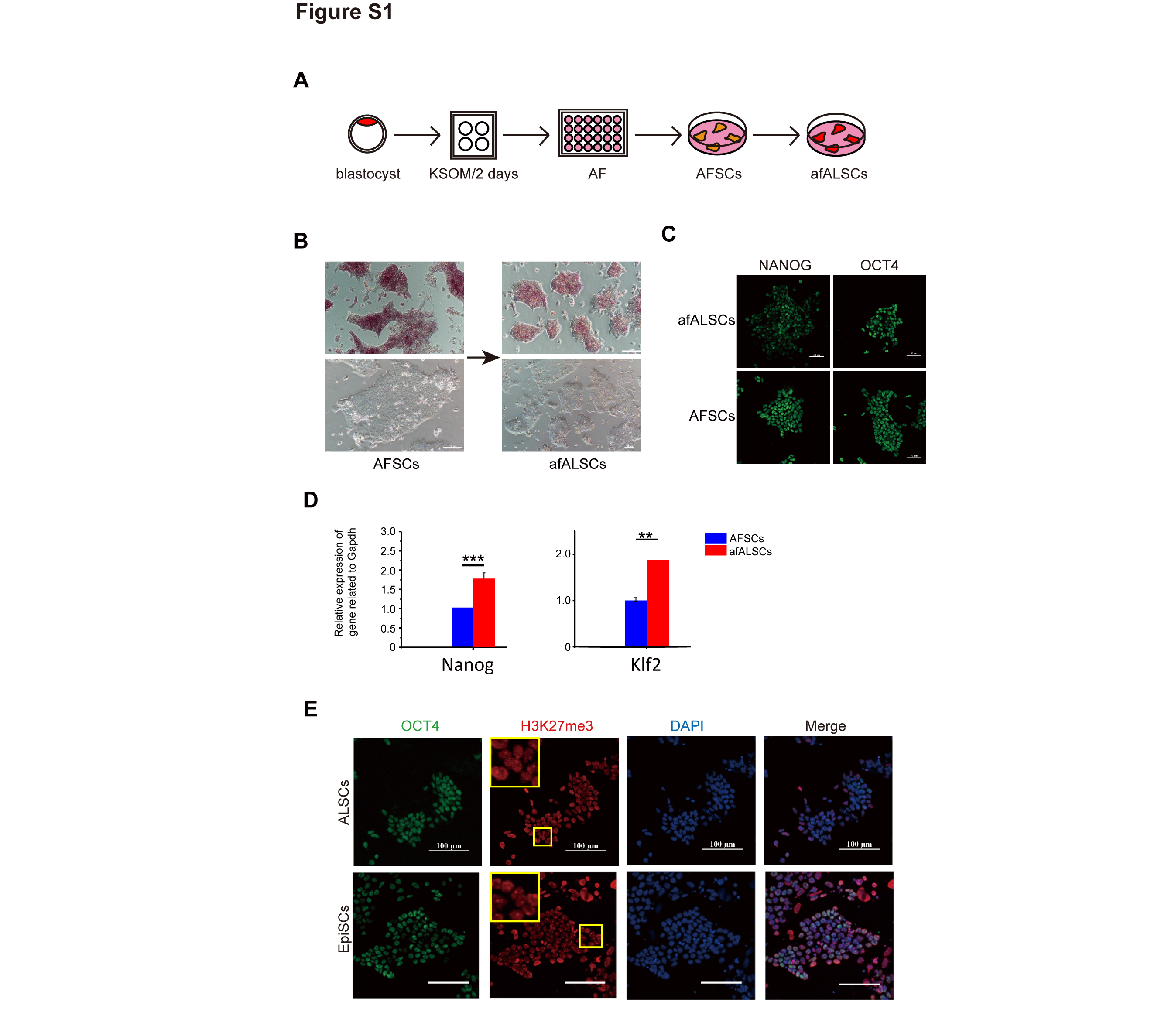

Supplement: Supplementary file 2 [file Image_1.tif]

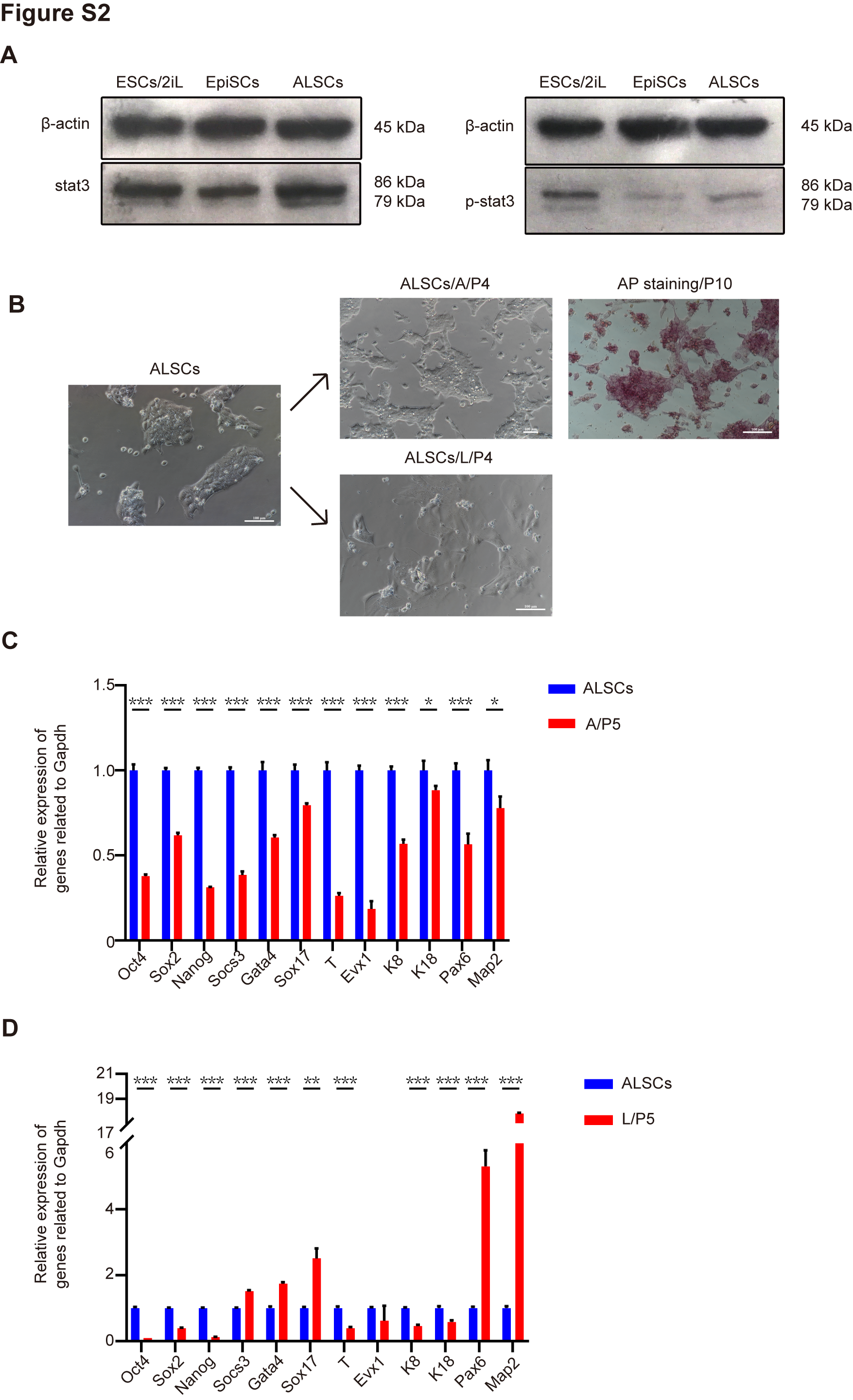

Supplement: Supplementary file 3 [file Image_2.TIF]

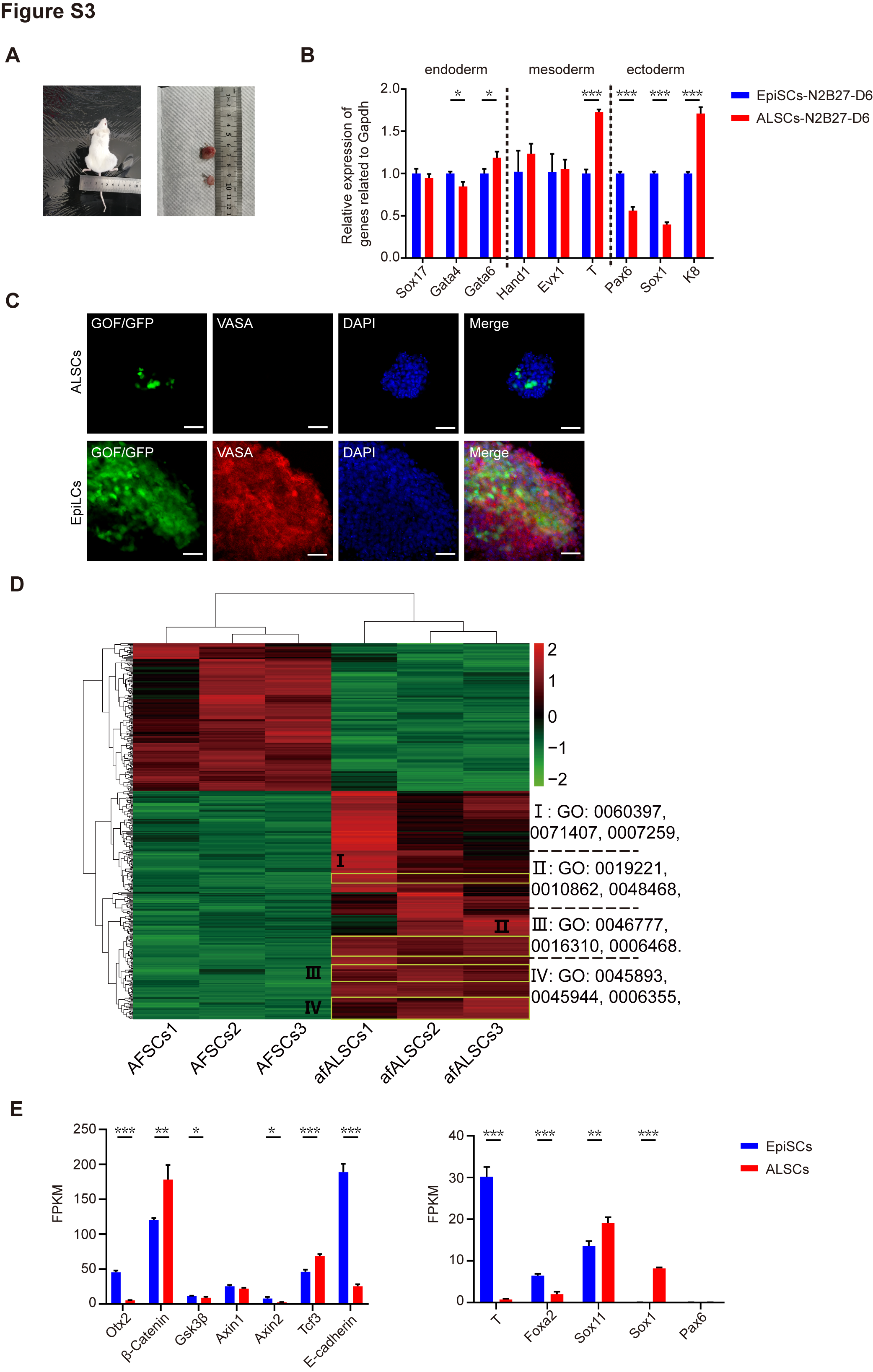

Supplement: Supplementary file 4 [file Image_3.TIF]

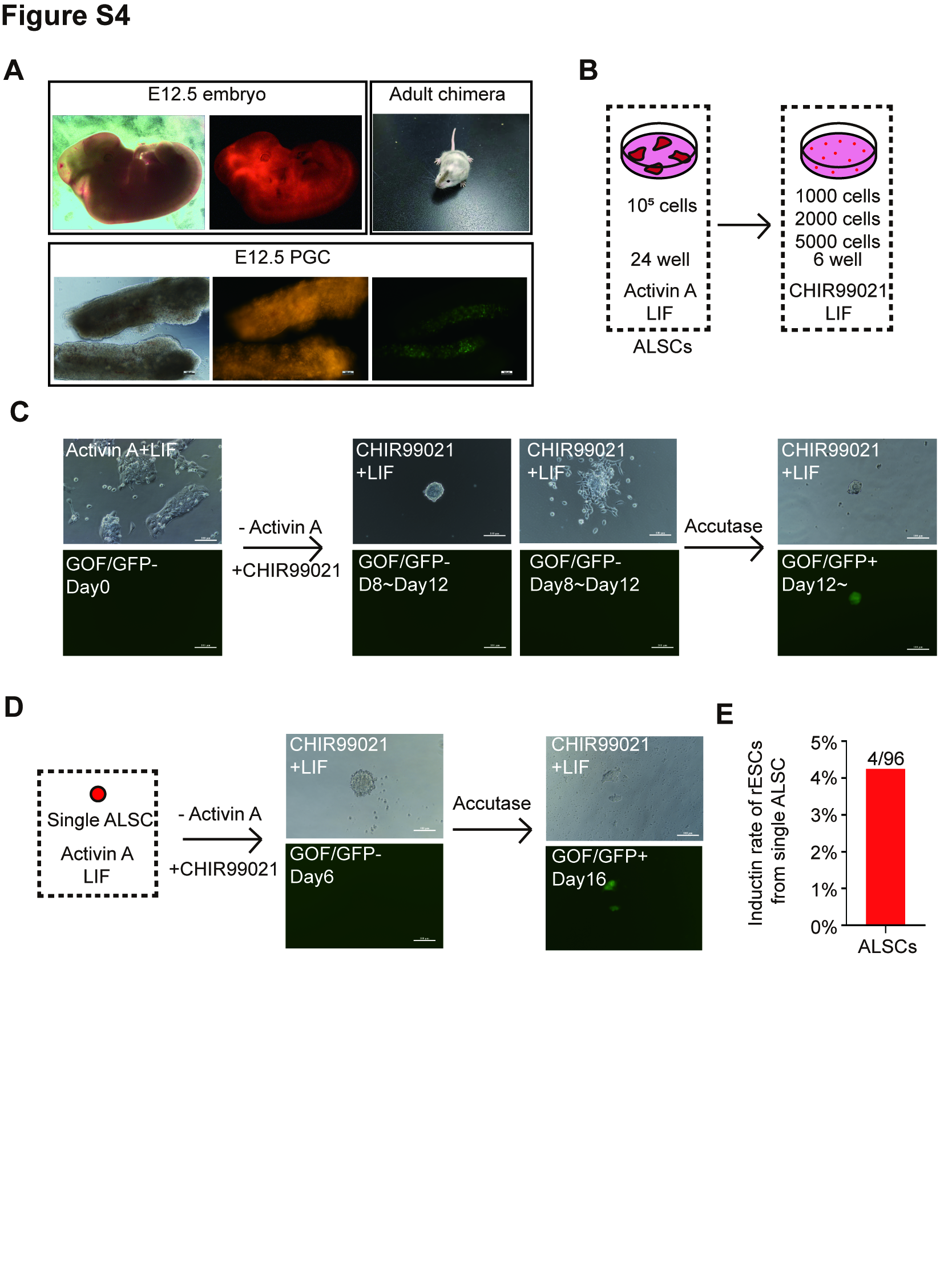

Supplement: Supplementary file 5 [file Image_4.TIF]
